# Computational models in genetics at BGRS-2018

**DOI:** 10.1186/s12863-019-0724-1

**Published:** 2019-03-18

**Authors:** Yuriy L. Orlov, Tatiana V. Tatarinova, Alex V. Kochetov

**Affiliations:** 1 Institute of Cytology and Genetics SB RAS, 630090 Novosibirsk, Russia; 2 Novosibirsk State University, 630090 Novosibirsk, Russia; 3 Department of Biology, University of La Verne, La Verne, CA USA; 4 Vavilov Institute for General Genetics, Moscow, Russia; 5 Siberian Federal University, Krasnoyarsk, Russia

This Special Issue of *BMC Genetics *assembles papers on genetics presented at the biannual summit in Bioinformatics and Systems Biology BGRS\SB (Bioinformatics of Genome Regulation and Structure\Systems Biology) – 2018 (http://conf.bionet.nsc.ru/bgrssb2018/en/). BGRS\SB-2018 was XI-th in the conference series in Novosibirsk since 1998. Genetics models as well as computational genomics were presented at the conference from the beginning and highlighted at BioMed Central special journal issues since 2014 (https://bmcgenomics.biomedcentral.com/articles/supplements/volume-15-supplement-12) [1–3]. It also included *BMC Genetics* Supplement (https://bmcgenet.biomedcentral.com/articles/supplements/volume-16-supplement-1) [4], selected papers in microbiology models (https://bmcmicrobiol.biomedcentral.com/articles/supplements/volume-16-supplement-1), structural biology (https://bmcstructbiol.biomedcentral.com/articles/supplements/volume-18-supplement-1). To accompany this Special Issue, other Supplements compiling selected BGRS-2018 articles in the fields of genomics, bioinformatics, plant biology, evolutionary biology and systems biology are published as a part of following journal series: *BMC Genomics, BMC Bioinformatics, BMC Systems Biology, BMC Medical Genomics, BMC Evolutionary Biology* and *BMC Plant Biology* [5–9]. In the past, respective highlights were organized into the Special Issues with reports from “Belyaev Readings-2017” memorial event (http://conf.bionet.nsc.ru/belyaev100/en) [10–15]. The Belyaev Conference-2017 (“Belyaev Readings - 2017”) was devoted to science legacy of outstanding scientist, evolutionist and geneticist, full member of the USSR Academy of Sciences, Professor Dmitry K. Belyaev (1917–1985) to mark the 100-th anniversary since his birth [16]. Such science problems include selection works on fox and rat lines [17], search for genes related to climate adaptation [18] including published earlier at *BMC Genetics* [4].

This year we have attracted presentations covering a variety of topics – from plant and animal genetics to climate adaptation. The best papers were selected for publication in this special issue, and the brief summary is presented below.

Igoshin et al. focused their research on the cold stress in Siberian cattle [19] continuing climate adaptation topic [18]. Siberia is notoriously known for its cold climate with long snowy winters and short summers. Design of new highly productive livestock breeds adapted to harsh climatic conditions is therefore an important aim of modern agriculture and breeding. However, our knowledge of the genetic mechanisms of adaptation to local environments is still scarce. To address this issue for cold climates Igoshin and colleagues used an integrated approach for detecting genomic intervals related to body temperature maintenance under acute cold stress. The team was able to detect a single candidate region on cattle chromosome 15 overlapping between the GWAS results and the results of scans for selective sweeps. This important finding will allow breeders to create cattle breeds thriving in the cold climate of Siberia as well as in other cold regions of the world.

Andreyeva et al. [20] continued the topic of fruit fly research, discussing the role dCNDP2 plays in G2/M transition of the cell cycle in *Drosophila melanogaster*. This work is another application of animal models for analysis of human disease. Expression of the CNDP2 gene is frequently up- or down-regulated in different types of human cancers. The authors demonstrated that one of the dCNDP2 isoforms is expressed throughout the different tissues tested, and detected in both the cytoplasm and the nucleus. The presented approach will allow further functional characterization of the conserved CNDP2 protein using *D. melanogaster* as a model system.

The next group of papers in dedicated to plant genetics. Glagoleva et al. [21] investigated the evolution of the chalcone synthase gene family in bread wheat and relative species. The authors investigated structural and functional organization of the chalcone synthase genes and discussed evolution of this gene family in bread wheat and relative species. Putative members of this family were examined using genomic and transcriptomic approaches, resulting in the final list of well-characterized eight genes. Transcriptional assay along with a comparative analysis of the cis-regulatory elements revealed their functional diversification. The multiple functions supported by the chalcone synthase family are assumed to be a driving force for duplications of the chalcone synthase gene and their retention in plant genome. Wheat genes analysis papers by the groups from the same institute were published at *BMC Genetics* [22] and *BMC Plant Biol* [23, 24] special post-conference issues including 2019 issue [25].

Strygina et al. [26] were interested in genetic control of anthocyanin pigmentation of potato tissues. Potato is one of the most important crops worldwide, and a main component in diets in many cultures worldwide. Anthocyanins synthesis and accumulation in potato tissues are considered as one of important traits related to stress resistance and nutritional value. Strygina and co-authors investigated the genetic control of pigmentation of different potato tissues and concluded that that StAN1 is the major regulatory gene controlling potato anthocyanin synthesis, however, other genes are also involved in formation of potato pigmentation patterns. This is the first step in developing of a pigmentation diagnostic assay. This work continues potato resistance analysis presented earlier at *BMC Plant Biol* special issue after Belyaev Conference - 2017 [27].

The final paper of this Special Issue, written by Khlestkin et al. [28] discusses potato GWAS to find starch phosphorylation associated SNPs. Potato tubers cannot be stored for long periods of time, therefore, the use of potatoes in the form of starch and products from starch modification appears as a reasonable alternative. To increase the appeal of potato starch as a feedstock for various industries, substantial attempts to improve its molecular composition and physical and chemical properties are being made. Physical and chemical properties of potato starch gels is dependent on its phosphate content; phosphorus is important for proper nutrition, and potatoes and starch high in phosphate would be valuable as food. Kleshkin et al. performed a genome-wide association study using a 22 K SNP potato array to find novel genomic regions associated with starch phosphorylation. The researchers found eight novel genomic regions associated with starch phosphorylation. This study will support selection of the most informative SNPs for developing convenient diagnostic markers to accelerate the breeding of potatoes with predetermined levels of starch phosphorylation.

We offer to our readers a wide range of reports describing recent breakthrough in genetics presented at the conference as well as science trends in related fields. Young scientists gathered in Novosibirsk for a School “Systems Biology and Bioinformatics” (SBB-2018) co-authored current issue and parallel Supplements 2019 [5–9]. In previous years, the materials of SBB Schools were published in Special Issues [29] including *BMC Genetics* [30]. Genomics field will be highlighted at VII Congress of Vavilov Society of Geneticists and Breeders organized by Saint Petersburg State University 18–22 June 2019 (https://events.spbu.ru/events/genetic-selection-2019). Soon after, 24–29 June 2019, 5th International scientific conference “Plant genetics, genomics, bioinformatics and biotechnology” (PlantGen2019) will be organized in Novosibirsk, Russia (http://conf.bionet.nsc.ru/plantgen2019/en/) focusing on plant genomics field together with SBB-2019 School. Next BGRS\SB-2020 event is scheduled to June 2020 in Novosibirsk, Russia. We invite our readers worldwide to attend our next events!
